# Psychological Safety and Workaholism Among Nurses: The Mediating Role of Patient Expectation Management and the Moderating Role of Age

**DOI:** 10.1155/jonm/1799704

**Published:** 2026-09-21

**Authors:** Selman Kizilkaya, Gülizar Gülcan Şeremet, Dilek Uslu

**Affiliations:** ^1^ Department of Health Management, Faculty of Economics and Administrative Sciences, Dicle University, Diyarbakır, Türkiye, dicle.edu.tr; ^2^ Department of Health Management, Faculty of Health Sciences, Çankırı Karatekin University, Çankırı, Türkiye, karatekin.edu.tr; ^3^ Department of Health Management, Faculty of Economics and Administrative Sciences, Ankara Hacı Bayram Veli University, Ankara, Türkiye

**Keywords:** age moderation, nursing workforce, occupational well-being, patient expectation management, psychological safety, workaholism

## Abstract

**Background:**

Psychological safety is an important organizational resource that promotes open communication, learning, and collaboration in nursing. However, its potential association with less desirable outcomes, such as workaholism, remains unclear. This study examined the associations between perceived psychological safety and workaholism among nurses, focusing on the mediating roles of supporting and suppressing patient expectations and the moderating role of age.

**Methods:**

Data for this study were obtained from 523 nurses employed in public hospitals across Türkiye using a cross‐sectional survey design. Data were collected using validated measures of perceived psychological safety, patient expectation management, and workaholism. Gender, marital status, professional experience, unit experience, and role were included as covariates. The hypothesized conditional process model was evaluated using Hayes’ PROCESS macro (Model 58), incorporating 5000 bootstrap samples and HC3 robust standard error estimates.

**Results:**

Perceived psychological safety was positively associated with supporting expectations (*b* = 0.762, *p* < 0.001) and suppressing expectations (*b* = 0.241, *p* < 0.001). Both dimensions were positively associated with workaholism. Significant indirect effects were observed through supporting expectations (effect = 0.170, 95% CI [0.057, 0.291]) and suppressing expectations (effect = 0.209, 95% CI [0.126, 0.292]) at mean age. Age moderated the association between psychological safety and suppressing expectations, with stronger effects among older nurses. Age also moderated the association between supporting expectations and workaholism, such that the indirect effect was significant only at mean and higher age levels.

**Conclusions:**

Perceived psychological safety may be associated with workaholism through patient expectation management behaviors, with age strengthening specific pathways. These findings highlight the importance of balancing psychologically safe work environments with strategies that promote sustainable work practices.

**Implications for Nursing Management:**

Nursing managers should complement psychological safety initiatives with workload monitoring, role clarity, boundary‐setting support, and patient expectation management training. Age‐sensitive workload and support policies may help reduce the risk of workaholism and promote nurse well‐being.

## 1. Introduction

Psychological safety describes a workplace climate in which individuals feel comfortable expressing ideas, raising concerns, asking questions, and acknowledging mistakes without anticipating negative interpersonal or professional repercussions [1, 2]. Initially emerging from organizational behavior research, psychological safety has evolved into a key framework for explaining communication, learning, and collaboration within complex healthcare teams [1]. In healthcare, where patient outcomes depend heavily on timely communication, teamwork, error reporting, and coordinated decision‐making, psychological safety has been consistently associated with learning behaviors, communication openness, patient safety practices, and favorable job outcomes [3–6]. Conceptual and empirical work in healthcare further suggests that psychological safety is not merely an individual perception but a relational and contextual condition shaped by team climate, nonpunitive culture, interpersonal trust, and organizational support [3, 4].

The importance of psychological safety is especially evident in nursing environments. Nurses work in high‐stakes settings characterized by heavy workloads, continuous patient contact, emotional labor, time pressure, and responsibility for patient well‐being. Under these conditions, psychologically safe environments may support open communication, collaborative problem‐solving, willingness to report concerns, and effective responses to clinical uncertainty [4–6]. Previous studies among nurses and nurse managers have shown that psychological safety is associated with communication openness, job satisfaction, commitment, social support, reduced turnover intention, and improved patient safety perceptions [5–8]. Therefore, psychological safety is commonly regarded as a valuable organizational resource that supports both employee well‐being and care quality.

However, the literature has focused predominantly on the beneficial outcomes of psychological safety, while its possible associations with less desirable work outcomes remain relatively underexplored. To better understand these relationships, the present study draws on job demands–resources (JD‐R) theory, which emphasizes the role of workplace resources in enabling employees to navigate occupational demands while fostering motivation, performance, and personal development [9–11]. Yet, in occupations marked by sustained emotional, cognitive, and relational demands, job resources may also be associated with stronger work investment, greater role involvement, and increased effort directed toward demanding work tasks [10, 11]. Within the JD‐R framework, workaholism has been conceptualized not merely as an outcome of job demands but as a personal demand—an internally driven, compulsive tendency to overwork that operates alongside and interacts with job‐level characteristics [12, 13]. In healthcare settings, where professional responsibility and patient‐centered expectations are intense, psychological safety may be associated not only with healthy engagement but also with excessive work investment under certain conditions.

One form of excessive work investment is workaholism. Workaholism is generally defined as a compulsive and excessive tendency to work, characterized by an internal drive to work beyond what is reasonably expected and by persistent preoccupation with work even outside working hours [14, 15]. Although workaholism and work engagement both involve high work investment, they are conceptually distinct. Work engagement reflects a positive motivational state involving vigor, dedication, and absorption, whereas workaholism reflects compulsive overinvestment that is often associated with impaired well‐being, emotional exhaustion, role overload, work–life imbalance, and adverse mental health outcomes [16–18]. This distinction is particularly important for interpreting the present findings: the same organizational resource that supports healthy engagement may, under high‐demand conditions, also be associated with compulsive overwork, especially when nurses internalize elevated performance expectations as personal obligations [12, 19].

The potential link between psychological safety and workaholism should therefore be interpreted carefully. This study does not argue that psychological safety is harmful or that it causes workaholism. Rather, it examines whether psychological safety is statistically associated with workaholism in a nursing context and whether this association operates through specific relational work behaviors. Drawing on JD‐R theory, social exchange theory, and self‐determination theory, psychologically safe environments may provide nurses with support, trust, autonomy, and confidence to invest more deeply in patient care [9, 20, 21]. In high‐demand healthcare settings, such investment may be associated with a heightened sense of responsibility, difficulty disengaging from work, and greater susceptibility to workaholic tendencies.

A potentially important mechanism in this relationship is the ability to manage patient expectations. Patient expectation management refers to nurses’ ability to navigate, regulate, and respond to patients’ expectations during care delivery. This ability includes two complementary dimensions: supporting expectations, which involves reinforcing realistic expectations and fostering positive patient relationships, and suppressing expectations, which involves redirecting unrealistic or clinically impractical expectations [22]. These behaviors are central to nursing practice because nurses frequently act as the main point of communication between patients, families, physicians, and healthcare institutions. However, expectation management also requires substantial emotional labor, interpersonal sensitivity, cognitive effort, and professional judgment [22–24].

From a JD‐R perspective, psychological safety can be understood as a job resource that enables proactive interpersonal behavior, whereas patient expectation management can be understood as a demanding relational activity requiring continuous emotional and cognitive investment [25–27]. Nurses who feel psychologically safe may be more willing and confident to engage in difficult patient interactions, provide reassurance, correct unrealistic expectations, and sustain therapeutic relationships. Although these behaviors are valuable for care quality, sustained involvement in expectation management may also be associated with higher work investment and workaholic tendencies. Despite this theoretical plausibility, research examining patient expectation management as a mediator between psychological safety and workaholism remains limited.

Age may further shape these associations. Lifespan and occupational aging research suggests that age is associated with differences in emotional regulation, coping strategies, occupational experience, professional identity, and responses to workplace demands [28–33]. Critically, chronological age is conceptually distinct from professional experience and organizational tenure, with which it is often correlated. Whereas professional experience and unit tenure reflect accumulated job‐specific knowledge, task familiarity, and organizational socialization, chronological age indexes broader lifespan developmental processes—including changes in emotional regulation competence, motivational orientation, and professional identity—that unfold independently of specific role history [34, 35]. Older employees often possess greater experience in handling emotionally charged interactions and may use more refined emotion‐regulation strategies when responding to interpersonal demands [29–31]. These age‐related differences in emotional regulation and professional identity formation are grounded in lifespan developmental theories such as socioemotional selectivity theory and the strength and vulnerability integration model, which predict systematic shifts in emotional goals, regulation strategies, and interpersonal investment with advancing chronological age—shifts that are not reducible to accumulated work experience alone [36, 37]. In nursing, these age‐related differences may influence how psychological safety is translated into patient expectation management behaviors and how such behaviors are associated with workaholism. For this reason, age was selected as the moderator variable in the present study rather than professional experience or unit tenure: age captures biologically and psychosocially driven developmental changes that are theoretically more pertinent to the emotion‐regulatory and identity‐based mechanisms hypothesized to condition the pathways under investigation [34–37]. Therefore, the present study examines age as a moderator of specific paths within the model rather than as a general moderator of all relationships.

While previous research has predominantly emphasized the beneficial consequences of psychological safety, the present study explores its association with workaholism, thereby extending the scope of psychological safety research to a relatively underexamined outcome. It also advances understanding of the mechanisms underlying this relationship by identifying supporting and suppressing patient expectations as parallel mediators linking psychological safety and workaholism among nurses. In addition, the study provides greater clarity regarding the role of age by specifying and testing the particular pathways through which age may shape these associations. By integrating psychological safety, patient expectation management, workaholism, and age within a JD‐R‐based moderated mediation framework, this study offers a more nuanced understanding of how beneficial organizational resources may be associated with both adaptive and potentially maladaptive forms of work investment in healthcare.

To distinguish lifespan developmental influences from experience‐related influences, professional experience and unit tenure were treated as control variables throughout the analyses, whereas chronological age was retained as the focal moderator based on its distinct theoretical relevance. This approach enabled the study to examine whether age explains variation in the proposed relationships beyond occupational experience and organizational tenure.

## 2. Theoretical Background and Hypotheses

### 2.1. The Effect of Perceived Psychological Safety (PPS) on Workaholism

The JD‐R framework provides the theoretical foundation for the present study. This perspective proposes that employees’ attitudes, behaviors, and well‐being are influenced by the dynamic interplay between work‐related demands and the resources available to address them [9–11]. Whereas job demands require sustained physical, emotional, or cognitive effort, job resources facilitate goal attainment, support employee development, and buffer the impact of workplace pressures [9]. Psychological safety can be conceptualized as a job resource because it reduces interpersonal risk, facilitates communication, encourages help‐seeking, and supports active participation in work processes [1, 3, 4].

Traditionally, psychological safety has been associated with beneficial outcomes such as learning, innovation, open communication, job satisfaction, commitment, and patient safety [3–8]. However, the JD‐R framework explicitly accommodates the possibility that job resources may also amplify work investment in ways that extend beyond healthy engagement. Schaufeli and Taris noted that within JD‐R theorizing, workaholism can be positioned as a personal demand—an internally driven requirement that individuals impose on themselves, which may be activated or intensified when supportive resources lower the perceived barriers to work involvement [13]. Guglielmi et al. demonstrated empirically that workaholism, as a personal demand, operates as an initiator of the health impairment process within the JD‐R model, contributing to burnout through elevated job demands [12]. These findings indicate that in environments where resources are abundant, the very conditions that reduce interpersonal risk and foster open participation may also create the conditions under which compulsive work patterns take hold. Contemporary JD‐R theorizing further suggests that job resources may stimulate greater work investment, especially when employees operate in environments with high emotional and relational demands [10, 11]. In nursing, psychological safety may be associated with greater willingness among nurses to assume more responsibility, participate more actively in patient care, communicate more openly, and invest greater effort in solving patient‐related problems. These behaviors are valuable, but they may also be associated with excessive work involvement when combined with intense role demands.

The distinction between work engagement and workaholism is essential for interpreting this association. Di Stefano and Gaudiino demonstrated that workaholism and work engagement, while both associated with high work investment, produce markedly different patterns of work–life interference, with workaholism uniquely and positively associated with work‐to‐life conflict [19]. Taris et al. similarly showed that workaholism is associated with increasing introjected regulation — that is, work driven by internal compulsion and avoidance of guilt rather than genuine interest — whereas work engagement is associated with more autonomous and intrinsically motivated forms of work involvement [16]. Accordingly, the positive association between psychological safety and workaholism, if confirmed, would not imply that psychological safety promotes healthy engagement but rather that it may, in certain high‐demand contexts, facilitate the conditions under which compulsive overwork emerges.

Social exchange theory provides a complementary explanation for this association. When nurses perceive their work environment as supportive and trusting, they may feel an obligation to reciprocate through increased effort, loyalty, and professional dedication [20]. In high‐demand healthcare contexts, such reciprocity may be expressed as persistent overinvestment in work. Self‐determination theory also suggests that psychologically safe environments may satisfy needs for autonomy, competence, and relatedness, thereby increasing motivation and role involvement [21].

Accordingly, it is expected that

 H1: PPS is positively associated with workaholism.

### 2.2. Psychological Safety and Patient Expectation Management

Patient expectation management is a core relational competency in nursing. Nurses must communicate care‐related information, provide emotional reassurance, address uncertainty, and respond to patients’ and families’ expectations. This competency includes two dimensions: supporting expectations and suppressing expectations [22]. Supporting expectations involves empathic communication, emotional validation, reassurance, and the reinforcement of realistic expectations. Suppressing expectations involves redirecting, limiting, or correcting unrealistic expectations that may not align with clinical or organizational realities.

Both dimensions require emotional labor, cognitive control, and interpersonal skill [22–24]. Psychological safety may enhance nurses’ ability to engage in these behaviors because it reduces fear of negative evaluation and supports open, confident communication [1, 3–5]. In psychologically safe environments, nurses may feel more able to initiate difficult conversations, clarify misunderstandings, and manage patient expectations without fear of blame or criticism.

Psychological safety may provide nurses with the confidence and support needed to engage more actively in patient‐related interactions, particularly those requiring effective expectation management [9–11]. Consistent with social exchange theory, supportive workplace environments may also encourage employees to invest greater effort in patient‐centered behaviors [20]. As a result, PPS is expected to be positively associated with both dimensions of patient expectation management.

Accordingly, the following hypotheses are advanced:

 H2a: PPS is positively associated with supporting patient expectations.
 H2b: PPS is positively associated with suppressing patient expectations.

### 2.3. Patient Expectation Management and Workaholism

Although patient expectation management is essential for high‐quality care, it may also increase nurses’ work investment. Supporting patient expectations requires sustained emotional engagement, reassurance, empathy, and the maintenance of therapeutic relationships. These behaviors may strengthen nurses’ sense of responsibility and indispensability, thereby making psychological disengagement from work more difficult.

Suppressing patient expectations requires a different but equally demanding form of effort. Nurses must manage unrealistic requests, communicate limitations, handle dissatisfaction, and maintain professional composure in emotionally sensitive situations. Such interactions can increase cognitive burden, emotional strain, and persistent work‐related rumination [22–24].

Research on workaholism suggests that excessive and compulsive work patterns are associated with high work demands, overcommitment, role overload, and impaired well‐being [14–18, 25–27]. In nursing, these risks may be especially relevant because relational responsibilities often extend beyond formal task completion. Consequently, both dimensions of patient expectation management may be associated with higher levels of workaholism.

Accordingly, the following hypotheses are proposed:

 H3a: supporting patient expectations is positively associated with workaholism.
 H3b: suppressing patient expectations is positively associated with workaholism.

### 2.4. The Mediating Role of Patient Expectation Management

Building on the previous arguments, patient expectation management is proposed as a mediator in the association between psychological safety and workaholism. Within the JD‐R framework, job resources may shape employee outcomes by enabling greater involvement in demanding work activities [9–11]. Psychological safety may encourage nurses to invest more effort in patient communication and expectation management. These behaviors, in turn, may be associated with greater emotional labor, cognitive load, work intensity, and difficulty disengaging from professional responsibilities.

The supporting expectations pathway reflects a mechanism through which psychological safety may encourage nurses to invest additional emotional and interpersonal effort in patient‐centered care, which may be associated with overcommitment when nurses internalize patient needs as personal responsibilities. The suppressing expectations pathway reflects a mechanism through which psychological safety may facilitate active management of unrealistic expectations, requiring substantial emotional regulation and cognitive effort.

Consequently, patient expectation management may represent an important behavioral mechanism through which psychological safety becomes associated with workaholism.

Accordingly, the following hypotheses are proposed:

 H4a: supporting patient expectations mediates the positive association between PPS and workaholism.
 H4b: suppressing patient expectations mediates the positive association between PPS and workaholism.

### 2.5. The Moderating Role of Age

Age is proposed as a boundary condition in the present model. As noted previously, chronological age captures lifespan developmental processes—including changes in emotional regulation competence, motivational orientation, socioemotional goal prioritization, and professional identity consolidation — that are conceptually and empirically distinct from professional experience and organizational tenure [34–37]. Lifespan development and occupational aging perspectives suggest that employees differ across age groups in emotional regulation, coping capacity, occupational experience, and professional identity [28–32]. Older workers often show stronger emotion‐regulation skills and greater experience in managing complex interpersonal situations [29, 30]. These age‐related differences may shape how nurses use psychological safety and how they respond to patient expectation management demands.

In the present model, age is hypothesized to moderate two specific pathways. First, age is expected to moderate the association between PPS and suppressing expectations. These pathways were selected on theoretical rather than statistical grounds. Age was not expected to moderate the relationship between PPS and supporting patient expectations because supportive patient communication represents a routine interpersonal behavior that is generally encouraged across all career stages when nurses perceive a psychologically safe environment [3–6]. Likewise, age was not hypothesized to moderate the association between suppressing patient expectations and workaholism because the emotional and cognitive demands associated with managing unrealistic patient expectations are expected to contribute to compulsive work investment regardless of employees’ developmental stage. Instead, age was expected to be particularly relevant in pathways involving emotion‐regulation competence and professional identity, both of which have been shown to undergo systematic developmental changes across adulthood [29–32, 38]. Older nurses may be more likely to translate psychological safety into suppressing behaviors because they possess greater experience in managing difficult patient interactions and communicating clinical limits. Scheibe and Moghimi showed that older workers tend to maintain more stable emotion‐regulation patterns in response to intense work events, suggesting that older nurses may more consistently recruit suppressive strategies when psychological safety provides a supportive context [31]. Thus, when psychological safety is high, older nurses may feel particularly able to redirect unrealistic expectations.

Second, age is expected to moderate the association between supporting expectations and workaholism. Older nurses may have stronger professional identities and deeper role commitment, which may make emotionally supportive patient interactions more closely linked with overcommitment. Livingstone and Isaacowitz demonstrated that older adults show stronger tendencies toward positivity‐enhancing and emotionally engaging regulatory behaviors in daily life, which in a professional context may translate into greater sustained investment in supportive patient care [38]. In contrast, younger nurses may engage in supporting behaviors without the same accumulated professional identity or responsibility burden. This expectation is broadly consistent with previous evidence suggesting that age conditions the mechanisms through which workaholism operates among healthcare professionals [39].

Accordingly, the following hypotheses are proposed:

 H5a: age moderates the association between PPS and suppressing patient expectations.
 H5b: age moderates the association between supporting patient expectations and workaholism.

## 3. Methodology

### 3.1. Research Model

The present research was designed to examine the associations among PPS, patient expectation management, and workaholism in nurses, while also exploring the potential moderating role of age. Although longitudinal and experimental designs are generally preferred for establishing causality, cross‐sectional designs are widely accepted for testing theoretically derived frameworks in exploratory research. Accordingly, the present study examined the hypothesized associations as an initial step toward understanding the underlying mechanisms linking these constructs. To enhance the credibility of the statistical inferences drawn from the data, robust standard error estimation (HC3) and bootstrap resampling procedures were incorporated into the analyses.

**FIGURE 1:**
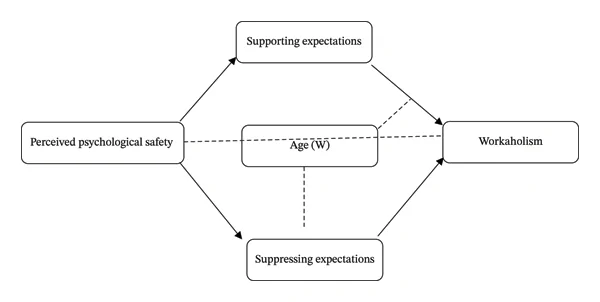
Conceptual diagram of the moderated mediation model (model 58).

In the proposed research model, PPS was conceptualized as the independent variable, and workaholism was the dependent variable. Supporting expectations and suppressing expectations were specified as parallel mediators, representing two distinct pathways through which psychological safety may be associated with workaholism. Age was hypothesized to moderate these mediation pathways. Consistent with lifespan developmental theory, age was selected as the moderator because it reflects developmental processes extending beyond accumulated occupational experience, including changes in emotion regulation, motivational orientation, and professional identity. By contrast, professional experience and tenure in the current unit primarily represent job‐specific knowledge and organizational familiarity. Therefore, professional experience and unit tenure were entered as covariates to statistically control their potential confounding influence, whereas age was retained as the theoretically relevant moderator. Thus, the strength of the indirect effects may vary across different age levels (Figure 1).

### 3.2. Study Group

The study was conducted with nurses recruited through convenience sampling, leveraging professional networks within hospitals and healthcare organizations across various regions in Türkiye. This recruitment approach allowed for the inclusion of nurses representing diverse roles and clinical settings, including unit managers, ward nurses, intensive care nurses, and outpatient clinic nurses. Nurses who were actively employed in public hospitals and who provided voluntary informed consent were eligible to participate. The final sample comprised 523 nurses. This sample size was considered adequate for moderated mediation analysis based on established guidelines recommending a minimum of 200 participants to ensure sufficient statistical power and reliable effect size estimation [40].

### 3.3. Measurement Tools

Data were gathered through a web‐based questionnaire administered via Google Forms. This approach enabled participation from nurses across different healthcare settings while ensuring anonymity and confidentiality. The questionnaire consisted of four sections. The first section elicited demographic information, including participants’ age, gender, marital status, professional experience, tenure in the current unit, and occupational role.

Workaholism was assessed using the Dutch work addiction scale (DUWAS), originally developed by Schaufeli et al. and subsequently adapted for Turkish populations by Doğan and Tel [15, 41]. The instrument contains eight items evaluated on a five‐point Likert scale ranging from 1 (“totally inappropriate”) to 5 (“totally appropriate”). Internal consistency was satisfactory in the present study (Cronbach’s *α* = 0.871).

Patient expectation management was measured using the ability to manage patient expectations scale developed by Miçooğulları et al. [22]. The scale comprises nine items grouped into two dimensions: supporting expectations and suppressing expectations. Responses are recorded on a five‐point Likert scale ranging from 1 (“strongly disagree”) to 5 (“strongly agree”). The overall scale demonstrated high internal consistency in the current sample (Cronbach’s *α* = 0.920).

PPS was evaluated using Edmondson’s psychological safety scale, adapted into Turkish by Bülbül et al. [1, 42]. The instrument consists of seven items rated on a five‐point Likert scale ranging from 1 (“strongly disagree”) to 5 (“strongly agree”). Consistent with the original scoring procedure, items 1, 3, and 5 were reverse coded prior to analysis. Reliability analysis indicated good internal consistency (Cronbach’s *α* = 0.859).

### 3.4. Ethics

The study protocol was reviewed and approved by the Social and Human Sciences Ethics Committee of Dicle University. Approval for conducting the surveys was granted through the 06.09.2024 decision, reference number 768994. All research procedures complied with internationally recognized ethical guidelines, including the Declaration of Helsinki. Participants received information regarding the study aims, procedures, and confidentiality safeguards before providing consent. Participation was voluntary, and individuals retained the right to withdraw from the study at any time without consequence.

### 3.5. Statistical Analysis

Statistical analyses were performed using IBM SPSS Statistics 26 and Hayes’ PROCESS macro (Version 4.2) [40]. Preliminary analyses included the calculation of descriptive statistics (mean, standard deviation, skewness, and kurtosis) to evaluate the distributional characteristics of the study variables. The internal consistency of each measure was examined using Cronbach’s alpha coefficients.

The proposed conditional process model was tested using PROCESS Model 58. Model 58 within the PROCESS framework specifies a moderated mediation structure in which a single moderator variable (W) simultaneously conditions both the a‐paths (from the predictor to each mediator) and the b‐paths (from each mediator to the outcome). This architecture allows the indirect effect of the predictor on the outcome—transmitted through each mediator—to vary as a function of the moderator, producing conditional indirect effects that can be estimated and compared across specified levels of the moderator [43]. In this model, PPS served as the predictor variable, workaholism as the outcome variable, supporting expectations (AMPES1) and suppressing expectations (AMPES2) as parallel mediators, and age as the moderator.

To reduce the influence of potential confounding factors, gender, marital status, professional experience, tenure in the current unit, and occupational role were entered as covariates. Prior to model estimation, all continuous variables were centered around their respective means to facilitate the interpretation of interaction effects and minimize potential multicollinearity. Robust HC3 standard errors were used throughout the analyses to provide more reliable parameter estimates in the presence of heteroscedasticity.

Indirect effects were evaluated using a bootstrap resampling procedure with 5000 iterations, and statistical significance was determined based on 95% bias‐corrected confidence intervals. Indirect effects were considered significant when the corresponding confidence interval excluded zero. Conditional effects were examined at three representative values of the moderator: one standard deviation below the mean, the mean value, and one standard deviation above the mean.

Potential multicollinearity among predictors was assessed using variance inflation factor (VIF) statistics. All VIF values remained below the commonly accepted threshold of 5, indicating that collinearity was unlikely to affect model estimation. In addition, the dataset was screened prior to analysis, and no missing observations were detected among the 523 completed responses.

## 4. Results

The majority of participants were female (83.2%), while 16.8% were male. Regarding marital status, 60.6% were married and 39.4% were single. In terms of professional experience, 64.8% had more than 5 years of experience, 14.5% had between one and 5 years, and 20.7% had less than 1 year. For unit‐level experience, 36.9% had one to 5 years in their current unit, 35.8% had more than 5 years, and 27.3% had less than 1 year. The largest occupational subgroup was ward nurses (31.0%), followed by intensive care nurses (19.3%), outpatient clinic nurses (11.7%), unit managers (9.6%), emergency room nurses (6.7%), hemodialysis nurses (3.3%), and other roles (18.5%). The mean age of participants was approximately 35 years (SD = 11.28).

### 4.1. Descriptive Statistics and Correlations

Skewness and kurtosis values for all variables fell within the acceptable range of ±2, indicating that the data did not substantially deviate from a normal distribution and were suitable for parametric analyses. The mean score for workaholism was 25.01 (SD = 7.03), for overall patient expectation management 31.78 (SD = 8.54), for supporting expectations 21.89 (SD = 6.39), for suppressing expectations 9.89 (SD = 2.57), and for PPS 22.81 (SD = 6.01).

Bivariate correlation analyses revealed that all study variables were significantly and positively intercorrelated (*p* < 0.01). Workaholism was positively associated with overall patient expectation management (*r* = 0.621), supporting expectations (*r* = 0.588), suppressing expectations (*r* = 0.602), and PPS (*r* = 0.573). PPS was strongly associated with overall patient expectation management (*r* = 0.777), supporting expectations (*r* = 0.757), and suppressing expectations (*r* = 0.699). Notably, the correlation between the overall patient expectation management scale and the supporting expectations subscale was very high (*r* = 0.982), and the correlation with the suppressing expectations subscale was also substantial (*r* = 0.883), indicating considerable overlap between the total scale and its subscales. Accordingly, the overall patient expectation management scale was not included as a separate predictor in the moderated mediation model to avoid multicollinearity; only the two subscales were retained as parallel mediators. To evaluate whether the observed relationships might be influenced by common method variance, Harman’s single‐factor procedure was applied. An exploratory factor analysis including all 24 study items was performed without rotation. The dominant factor accounted for 44.63% of the overall variance, falling below the benchmark commonly used to indicate serious common method concerns. Accordingly, common method variance was unlikely to have materially affected the study findings (Table 1).

**TABLE 1:** Descriptive statistics and intercorrelations among study variables.

Variable	*M*	SD	Skewness	Kurtosis	1	2	3	4	5
1. Workaholism	25.01	7.03	−0.52	−0.47	—				
2. Patient expectation management	31.78	8.54	−0.89	−0.23	0.621^∗∗^	—			
3. Supporting expectations	21.89	6.39	−0.94	−0.21	0.588^∗∗^	0.982^∗∗^	—		
4. Suppressing expectations	9.89	2.57	−0.49	−0.07	0.602^∗∗^	0.883^∗∗^	0.778^∗∗^	—	
5. Perceived psychological safety	22.81	6.01	−0.19	−0.80	0.573^∗∗^	0.777^∗∗^	0.757^∗∗^	0.699^∗∗^	—

^∗∗^ *p* < 0.01.

### 4.2. Effects of PPS on the Mediators

The overall model predicting supporting expectations was statistically significant (*R* ^2^ = 0.614, F(HC3) = 98.631, *p* < 0.001). PPS was a significant positive predictor of supporting expectations (*b* = 0.762, SE = 0.033, *t* = 22.941, *p* < 0.001, 95% CI [0.696, 0.827]). The interaction between PPS and age (PPS × Age) was not statistically significant (*b* = 0.001, *p* = 0.637), indicating that age did not moderate the association between PPS and supporting expectations.

The overall model predicting suppressing expectations was also statistically significant (*R* ^2^ = 0.611, *F* (HC3) = 120.643, *p* < 0.001). PPS significantly and positively predicted suppressing expectations (*b* = 0.241, SE = 0.013, *t* = 17.915, *p* < 0.001, 95% CI [0.215, 0.268]). The PPS × age interaction was statistically significant (*b* = 0.004, SE = 0.001, *t* = 3.889, *p* < 0.001, 95% CI [0.002, 0.007], Δ*R* ^2^ = 0.011), indicating that age moderated this association. Probing the interaction at −1SD, mean, and +1SD of age revealed that the positive association between PPS and suppressing expectations strengthened with increasing age: *b* = 0.191 (SE = 0.019, *p* < 0.001) at low age, *b* = 0.241 (SE = 0.013, *p* < 0.001) at mean age, and *b* = 0.292 (SE = 0.018, *p* < 0.001) at high age (Table 2).

**TABLE 2:** Results of moderated mediation analysis: Outcomes of supporting and suppressing expectations.

Predictor	Supporting expectations			Suppressing expectations		
	*b*	SE (HC3)	95% CI	*b*	SE (HC3)	95% CI
Constant	−0.125	1.424	[−2.922, 2.672]	1.163	0.550	[0.083, 2.243]
PPS	0.762	0.033	[0.696, 0.827]	0.241	0.013	[0.215, 0.268]
Age	−0.059	0.027	[−0.111, −0.006]	0.018	0.012	[−0.006, 0.042]
PPS × age	0.001	0.003	[−0.004, 0.007]	0.004	0.001	[0.002, 0.007]
Gender	0.664	0.489	[−0.296, 1.625]	−0.110	0.202	[−0.508, 0.287]
Marital status	−0.306	0.430	[−1.151, 0.539]	0.033	0.192	[−0.345, 0.411]
Professional exp.	−1.781	0.381	[−2.530, −1.031]	−1.485	0.170	[−1.820, −1.150]
Unit exp.	1.302	0.397	[0.523, 2.081]	0.842	0.154	[0.541, 1.144]
Role	0.267	0.100	[0.070, 0.464]	0.232	0.041	[0.152, 0.313]
*R* ^2^	0.614			0.611		
*F* (HC3)	98.631			120.643		

**FIGURE 2:**
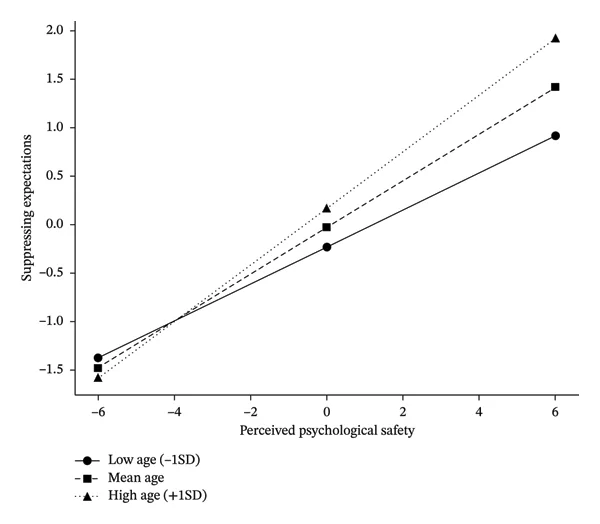
Moderating effect of age on the relationship between perceived psychological safety and suppressing expectations.

The moderating effect of age on the association between PPS and suppressing expectations is illustrated in Figure 2. As age increased from low to high levels, the slope of the relationship between PPS and suppressing expectations became progressively steeper, indicating that older nurses showed a stronger tendency to engage in suppressing behaviors as psychological safety increased (Figure 2).

### 4.3. Effects of the Mediators on Workaholism

The overall model predicting workaholism was statistically significant (*R* ^2^ = 0.442, F (HC3) = 43.700, *p* < 0.001). Both supporting expectations (*b* = 0.224, SE = 0.080, *t* = 2.813, *p* = 0.005, 95% CI [0.067, 0.380]) and suppressing expectations (*b* = 0.864, SE = 0.178, *t* = 4.866, *p* < 0.001, 95% CI [0.515, 1.213]) were significant positive predictors of workaholism. The direct effect of PPS on workaholism also remained significant after accounting for the mediators (*b* = 0.216, SE = 0.073, *t* = 2.959, *p* = 0.003, 95% CI [0.073, 0.360]), indicating partial mediation.

The interaction between supporting expectations and age (AMPES1 × age) was statistically significant (*b* = 0.012, SE = 0.005, *t* = 2.149, *p* = 0.032, Δ*R* ^2^ = 0.005), indicating that age moderated the association between supporting expectations and workaholism. Conditional effects revealed that this association was nonsignificant at low age (*b* = 0.092, SE = 0.111, *p* = 0.408, 95% CI [−0.127, 0.311]), but became significant at mean age (*b* = 0.224, SE = 0.080, *p* = 0.005, 95% CI [0.067, 0.380]) and high age (*b* = 0.355, SE = 0.088, *p* < 0.001, 95% CI [0.182, 0.528]). In contrast, the interaction between suppressing expectations and age (AMPES2 × age) was not statistically significant (*b* = −0.009, *p* = 0.447) (Table 3).

**TABLE 3:** Results of moderated mediation analysis: outcome of workaholism.

Predictor	*b*	SE (HC3)	*t*	*p*	95% CI
Constant	30.750	1.979	15.541	0.000	[26.863, 34.637]
PPS	0.216	0.073	2.959	0.003	[0.073, 0.360]
Supporting Exp. (AMPES1)	0.224	0.080	2.813	0.005	[0.067, 0.380]
Suppressing Exp. (AMPES2)	0.864	0.178	4.866	0.000	[0.515, 1.213]
Age	0.027	0.036	0.758	0.449	[−0.043, 0.097]
AMPES1 × age	0.012	0.005	2.149	0.032	[0.001, 0.022]
ampes2 × age	−0.009	0.012	−0.760	0.447	[−0.032, 0.014]
Gender	−1.041	0.670	−1.555	0.121	[−2.357, 0.275]
Marital status	−1.450	0.608	−2.384	0.018	[−2.646, −0.255]
Professional exp.	0.215	0.618	0.348	0.728	[−1.000, 1.430]
Unit exp.	−0.958	0.503	−1.904	0.057	[−1.947, 0.030]
Role	−0.081	0.125	−0.651	0.515	[−0.327, 0.164]
*R* ^2^	0.442				
*F* (HC3)	43.700			0.000	

**FIGURE 3:**
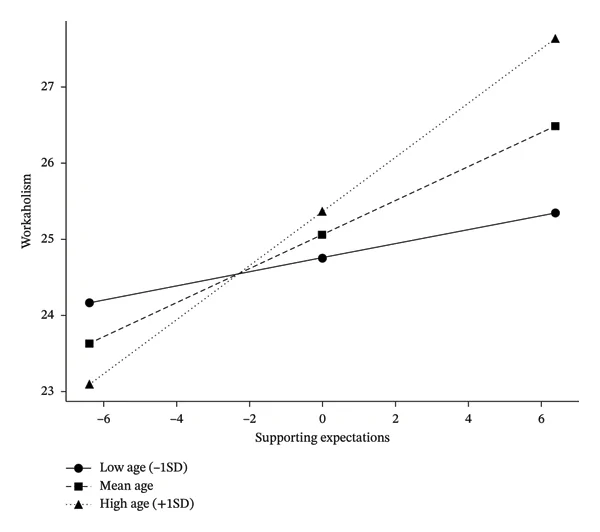
Moderating effect of age on the relationship between supporting expectations and workaholism.

The moderating effect of age on the association between supporting expectations and workaholism is illustrated in Figure 3. Among younger nurses, the relationship between supporting expectations and workaholism was relatively flat and nonsignificant, whereas among nurses at mean and higher age levels, this association became increasingly pronounced, suggesting that older nurses were more susceptible to workaholic tendencies when engaging in patient‐supporting behaviors (Figure 3).

### 4.4. Conditional Indirect Effects

Conditional indirect effects were estimated using percentile bootstrapping with 5000 resamples. For the pathway through supporting expectations (PPS ⟶ AMPES1 ⟶ WE), the indirect effect was nonsignificant at low age (effect = 0.069, BootSE = 0.082, 95% CI [−0.092, 0.239]), but statistically significant at mean age (effect = 0.170, BootSE = 0.060, 95% CI [0.057, 0.291]) and high age (effect = 0.276, BootSE = 0.066, 95% CI [0.152, 0.409]). Pairwise contrasts confirmed that the indirect effect at the mean age was significantly larger than the effect at low age (contrast = 0.102, 95% CI [0.017, 0.195]), and the indirect effect at high age was significantly larger than both low age (contrast = 0.207, 95% CI [0.033, 0.393]) and mean age (contrast = 0.106, 95% CI [0.016, 0.199]).

For the pathway through suppressing expectations (PPS ⟶ AMPES2 ⟶ WE), the indirect effect was statistically significant across all age levels: low age (effect = 0.184, BootSE = 0.047, 95% CI [0.095, 0.282]), mean age (effect = 0.209, BootSE = 0.042, 95% CI [0.126, 0.292]), and high age (effect = 0.223, BootSE = 0.058, 95% CI [0.108, 0.337]). Pairwise contrasts indicated no statistically significant differences between age groups for this pathway, suggesting that the indirect effect through suppressing expectations was relatively stable across age levels (Table 4).

**TABLE 4:** Conditional indirect effects of perceived psychological safety on workaholism.

Pathway	Age level	Effect	BootSE	95% CI	Significant
PPS ⟶ supporting Exp. ⟶ workaholism	Low (−1SD)	0.069	0.082	[−0.092, 0.239]	No
	Mean	0.170	0.060	[0.057, 0.291]	Yes
	High (+1SD)	0.276	0.066	[0.152, 0.409]	Yes

PPS ⟶ suppressing Exp. ⟶ workaholism	Low (−1SD)	0.184	0.047	[0.095, 0.282]	Yes
	Mean	0.209	0.042	[0.126, 0.292]	Yes
	High (+1SD)	0.223	0.058	[0.108, 0.337]	Yes

## 5. Discussion

The present study examined the associations among PPS, patient expectation management, and workaholism among nurses, while also investigating the moderating role of age. The findings indicated that PPS was positively associated with both supporting and suppressing patient expectations and that both dimensions were positively associated with workaholism. Furthermore, patient expectation management emerged as a significant mediating mechanism linking psychological safety and workaholism. The results highlight age as a relevant contextual factor shaping the relationships examined in this study. More specifically, older nurses exhibited stronger associations between psychological safety and suppressing expectations, as well as between supporting expectations and workaholism.

The positive association observed between PPS and workaholism may initially appear counterintuitive because psychological safety is generally regarded as a beneficial workplace resource associated with communication openness, trust, collaboration, employee well‐being, and patient safety [3, 44]. However, previous research on heavy work investment suggests that favorable workplace conditions do not necessarily preclude excessive involvement in work. Workaholism has been conceptualized as a form of heavy work investment characterized by excessive and compulsive working rather than enjoyment of work itself [16, 41, 45]. Importantly, Taris et al. reported that workaholism was associated with lower subsequent intrinsic motivation and higher levels of introjected regulation, suggesting that workaholic employees increasingly rely on internal pressures related to self‐worth and obligation rather than genuine enjoyment of work [16]. This finding indicates that excessive work involvement may persist even in environments that provide supportive conditions. Within the JD‐R framework, Guglielmi et al. demonstrated that workaholism, positioned as a personal demand, can be initiated and sustained independently of job‐level resources, as it stems from internally driven compulsions rather than external organizational requirements [12]. This conceptualization helps reconcile the apparent paradox: psychological safety as a job resource may simultaneously support healthy communication and engagement while also lowering the perceived barriers to overcommitment among nurses who are already predisposed toward compulsive work investment.

The distinction between workaholism and work engagement is particularly important when interpreting the present findings. Although both constructs involve substantial work investment, previous studies have emphasized that engaged employees work hard because they experience vigor, dedication, and enjoyment, whereas workaholic employees are driven by an internal compulsion to work [16, 41]. Similarly, Langseth‐Eide demonstrated that work engagement and workaholism represent distinct forms of heavy work investment with markedly different implications for employee well‐being and health outcomes [17]. Di Stefano and Gaudiino further showed that workaholism and work engagement produce opposite patterns of work–life interference: workaholism was more strongly associated with work‐to‐life conflict, whereas work engagement was more strongly associated with reduced life‐to‐work interference, underscoring that these constructs differ not only in their motivational basis but also in their consequences for nurses’ broader occupational well‐being [19]. Consequently, greater involvement in work‐related activities should not automatically be interpreted as evidence of healthy engagement. One possible interpretation of the present findings is that psychologically safe nurses may become increasingly invested in patient care and professional responsibilities, which may, under certain circumstances, become associated with excessive work involvement.

The positive associations between psychological safety and both dimensions of patient expectation management are broadly consistent with previous research on psychological safety in healthcare settings. Ito et al. described psychological safety as a condition that facilitates interpersonal risk‐taking behaviors, including speaking up, asking questions, expressing concerns, and engaging in open communication [3]. These behaviors are particularly relevant for patient expectation management because nurses frequently need to communicate realistic treatment outcomes, clarify misunderstandings, and negotiate potentially sensitive issues with patients and their families. In addition, trust, respect, effective communication, and nonpunitive workplace cultures have been identified as core attributes of psychologically safe healthcare environments [3]. Such characteristics may enable nurses to engage more confidently in both supporting and suppressing patient expectations. Therefore, the present findings support the view that psychological safety functions not only as a patient‐safety resource but also as an interpersonal resource that may facilitate challenging nurse–patient interactions.

Both supporting and suppressing patient expectations were positively associated with workaholism. These findings may be understood in light of the substantial emotional and cognitive demands involved in expectation management. Supporting patient expectations often requires continuous emotional availability, empathy, and relationship‐building, whereas suppressing unrealistic expectations requires emotional regulation, conflict management, and the communication of difficult information. Previous literature on heavy work investment suggests that sustained involvement in demanding interpersonal activities may contribute to stronger psychological investment in work [16, 17, 41, 45]. The present findings therefore suggest that patient expectation management may represent an important behavioral mechanism through which nurses become increasingly invested in their work roles.

The mediation findings further contribute to the literature by identifying patient expectation management as a potential pathway linking psychological safety and workaholism. Previous conceptualizations of psychological safety have largely emphasized positive outcomes such as learning, collaboration, work engagement, empowerment, and organizational commitment [3, 45]. The present findings extend this perspective by suggesting that psychological safety may also be associated with greater involvement in demanding patient‐centered behaviors, which may subsequently be related to excessive work investment. Importantly, this interpretation does not imply that psychological safety is harmful. Rather, it suggests that workplace resources may have complex associations with employee behavior, particularly in professions characterized by intensive interpersonal demands and strong caregiving norms.

The moderating role of age identified in the present study is consistent with previous occupational aging research. Scheibe reported that older employees experienced less disruption in attentional focus and end‐of‐day well‐being following highly negative work events compared with younger employees [30]. These age‐related differences were explained by more effective emotion‐regulation processes among older workers [30]. This perspective may help explain why the association between psychological safety and suppressing patient expectations was stronger among older nurses. Suppressing unrealistic expectations often requires emotional control, interpersonal sensitivity, and confidence in managing difficult interactions. Older nurses may be particularly effective in performing these tasks because accumulated professional experience and enhanced emotion‐regulation competencies enable them to navigate challenging patient encounters more effectively. These findings are consistent with Scheibe and Moghimi’s observation that older workers tend to maintain more stable emotion‐regulation patterns even under high‐intensity work conditions, suggesting that age‐related regulatory stability may translate into a stronger and more consistent tendency to deploy suppressive strategies when organizational safety conditions are favorable [31].

Age also strengthened the association between supporting patient expectations and workaholism. One possible explanation is that experienced nurses may become more deeply invested in patient‐centered activities because of stronger professional identities and greater commitment to caregiving responsibilities. Previous occupational aging research suggests that older employees often demonstrate greater emotional stability and resilience when confronting workplace challenges [30]. Although such strengths may facilitate effective patient care, they may also contribute to sustained involvement in emotionally meaningful work activities. Consequently, supportive patient interactions may become more strongly linked with excessive work investment among older nurses than among their younger counterparts. Livingstone and Isaacowitz’s experience‐sampling findings further support this interpretation: older adults were more likely to use positivity‐enhancing and emotionally engaging regulatory tactics in daily life, which in a professional caregiving context may translate into deeper and more sustained investment in supportive patient interactions, rendering older nurses more susceptible to workaholic overcommitment through this pathway [38].

Although age significantly moderated the a‐path from PPS to suppressing expectations (H5a supported), the conditional indirect effect through this suppressing expectations pathway did not differ significantly across age groups. This apparent inconsistency is theoretically interpretable within the moderated mediation framework. When a moderator conditions only the a‐path while the b‐path remains invariant across moderator levels, the magnitude of variation in the indirect effect is constrained by the relatively stable b‐path coefficient [43]. In the present study, the b‐path from suppressing expectations to workaholism did not vary significantly with age (AMPES2 × age interaction: *b* = −0.009, *p* = 0.447), meaning that although older nurses showed a stronger association between psychological safety and suppressing behaviors, this did not translate into proportionally larger differences in workaholic outcomes across age groups, as the subsequent link from suppressing behaviors to workaholism remained comparably strong across all age levels. This pattern suggests that suppressing expectations is robustly associated with workaholism regardless of age, whereas the sensitivity of this pathway’s activation to psychological safety is age‐dependent.

The present findings should also be interpreted in light of the broader literature on workaholism. Recent evidence indicates that workaholism is associated with a wide range of adverse occupational and health outcomes, including burnout, occupational stress, anxiety, depression, sleep disturbances, impaired concentration, and negative work‐related incidents [25, 26]. Among nursing professionals specifically, workaholism has been linked to emotional exhaustion, depersonalization, reduced professional effectiveness, and diminished quality of care [25]. These findings underscore the importance of identifying organizational and interpersonal mechanisms associated with excessive work investment. Although patient expectation management is an essential component of high‐quality nursing care, the present results suggest that sustained investment in emotionally demanding patient interactions may be associated with workaholic tendencies when not accompanied by adequate recovery opportunities and professional boundaries.

Regarding the intercorrelation between supporting and suppressing expectations (*r* = 0.778), Rönkkö and Cho note that a correlation of this magnitude does not in itself constitute evidence of a discriminant validity problem when the two constructs are conceptually distinguishable and when measurement error is appropriately considered [46]. The supporting and suppressing dimensions of the ability to manage patient expectations scale are conceptually distinct—the former involves empathic reinforcement of realistic expectations, whereas the latter involves the active redirection of unrealistic demands—and their separation is supported by the original scale development and validation [22]. In addition, VIF values for all predictors remained below the commonly recommended threshold, suggesting that multicollinearity was unlikely to have distorted the parameter estimates. Accordingly, although the correlation between the two subdimensions was relatively high, the available psychometric and statistical evidence supports their treatment as theoretically and empirically distinguishable constructs within the present model.

## 6. Conclusion

This study examined the associations among PPS, patient expectation management, and workaholism among nurses, with age as a moderating variable. The findings suggest that PPS is positively associated with workaholism and that the association was statistically accounted for in part by supporting and suppressing patient expectations. Age moderated specific pathways in the model, with older nurses showing stronger associations between psychological safety and suppressing expectations and between supporting expectations and workaholism.

These findings highlight that psychological safety, while broadly recognized as a beneficial organizational resource that fosters communication, learning, and collaboration, may also be associated with increased work investment and overcommitment under certain conditions. This does not imply that psychological safety is inherently harmful; rather, the findings suggest that organizations should complement efforts to enhance psychological safety with strategies that support boundary‐setting, workload monitoring, and sustainable work practices.

Patient expectation management emerged as a relevant mechanism linking psychological safety and workaholism, underscoring the importance of equipping nurses with the skills to navigate emotionally demanding patient interactions without excessive self‐sacrifice. Age‐sensitive approaches to workload distribution and professional support may also be warranted, given the differential moderating effects observed across age groups.

Given the cross‐sectional and self‐reported nature of the data, these conclusions should be treated as preliminary. Longitudinal and experimental studies are needed to establish the temporal ordering of these associations and to test the effectiveness of organizational interventions targeting the mechanisms identified in this study.

## 7. Practical Implications

The findings of this study carry several implications for nursing management and healthcare organizations. Healthcare administrators may consider developing structured training programs focused on patient expectation management, incorporating both supporting and suppressing dimensions, to help nurses regulate emotional labor demands and maintain professional boundaries. Such programs could support nurses’ capacity to manage patient interactions without excessive emotional or cognitive investment. Given that the association between supporting expectations and workaholism was more pronounced among older nurses, age‐sensitive approaches to workload distribution and professional development may be particularly relevant. Younger nurses may benefit from mentoring and boundary‐setting guidance, while older nurses may benefit from workload flexibility and opportunities for reflective practice.

Nursing managers should also be attentive to early signs of overcommitment, such as persistent overtime, emotional exhaustion, or difficulty disengaging from work, and be prepared to offer timely support. Organizational policies that institutionalize work‐life balance frameworks, including adequate rest periods, staffing rotations, and supportive supervision, may help sustain nurses’ occupational well‐being. These strategies are broadly consistent with the principles underlying Sustainable Development Goal 3 (good health and well‐being) in promoting a healthy and sustainable nursing workforce.

It should be emphasized that the present findings do not suggest that psychological safety itself is harmful. Rather, the results indicate that psychologically safe environments may benefit from complementary structural safeguards to prevent the unintended association between safety and overcommitment.

## 8. Limitations

While the study offers important insights into the relationships among psychological safety, patient expectation management, and workaholism, several limitations should be acknowledged. The cross‐sectional design precludes causal inferences regarding the temporal sequence of associations among psychological safety, patient expectation management, and workaholism. Cross‐sectional mediation analysis is known to produce potentially biased estimates [47], and the observed indirect effects should be interpreted as associational rather than causal. Future studies employing longitudinal or experimental designs would allow for stronger conclusions regarding the directionality of these relationships.

The exclusive inclusion of nurses from Türkiye may restrict the extent to which the findings can be transferred to other healthcare professions or cultural settings. Future research involving participants from different countries, healthcare systems, and professional groups would help determine whether the observed relationships remain consistent across diverse organizational and cultural environments.

Another limitation concerns the reliance on self‐reported measures for all study variables. Although the assessment of common method variance did not indicate a substantial threat to the findings, the possibility of response‐related biases, including social desirability and shared method effects, cannot be entirely excluded. Subsequent studies may benefit from incorporating objective indicators, supervisor evaluations, or data obtained from multiple sources to enhance the robustness of the findings.

Finally, while several demographic characteristics were statistically controlled, other workplace factors that may influence psychological safety, patient expectation management, and workaholism were beyond the scope of the present investigation. Variables such as leadership practices, team dynamics, organizational culture, and institutional characteristics may provide additional insight into the mechanisms underlying these relationships and should be considered in future research.

## Author Contributions

Selman Kizilkaya conceived and designed the study, conducted the data analysis, and drafted the main manuscript text.

Gülizar Gülcan Şeremet contributed to the development of the research design, data collection, visualization, and critical review of the manuscript.

Dilek Uslu assisted in data collection, methodological refinement, and manuscript editing.

All authors contributed to the interpretation of the findings, reviewed the final version of the manuscript, and approved it for submission.

## Funding

This study was supported by the Dicle University Scientific Research Projects Coordination Unit (DÜBAP), Project No: İİBF.26.009.

## Ethics Statement

The study was approved by the Dicle University Social and Human Sciences Ethics Committee on September 6, 2024 (approval number: 768994). Informed consent was obtained from all participants included in the study. The research was conducted in full accordance with the ethical standards set by the Declaration of Helsinki.

## Consent

Please see the Ethics Statement.

## Conflicts of Interest

The authors declare no conflicts of interest.

## Data Availability

The data that support the findings of this study are available from the corresponding author upon request.
